# How should comparative health reporting be designed in order to make it readily understandable? Thoughts and ideas to develop a health barometer for the Baden-Württemberg Health Map

**DOI:** 10.17886/RKI-GBE-2017-097

**Published:** 2017-08-30

**Authors:** Anne Würz

**Affiliations:** Ministry of Social Affairs and Integration, Baden-Württemberg Department 51 (Fundaments, Prevention, Public Health Service)

## Abstract

Baden-Württemberg’s Ministry for Social Affairs and Integration commissioned the Mannheim Institute for Public Health to develop an instrument which would enable health comparisons based on an analysis of strengths and weaknesses at the federal and municipal levels. This health barometer focuses on four key areas (population health, health promotion and disease prevention, health behaviour as well as out- and inpatient care), which are operationalised based on twelve indicators. The article explains how the individual indicators and the overall health barometer are calculated.

## Background

Activities to further develop health care in Baden-Württemberg are described in the state’s concept for the future of health care (the Zukunftsplan Gesundheit) [1]. Participation plays an important role in the concept, which means that besides establishing processes directed at participation (for example, in the development of guiding principles), the provision of information, such as data and health care sector facts, in adequate form becomes necessary [2].

Since 2011, a state health map has supported municipal level health care reporting by providing well-prepared information on the situation in the health care sector [3]. Cartographic comparisons in the health map highlight regional differences, which can serve as a basis for further discussion. To ensure the comparability between administrative and municipal districts regarding strengths and weaknesses in the health care sector, the Ministry for Social Affairs and Integration commissioned the Mannheim Institute for Public Health to develop its health barometer. A ranking aims to make regional needs identifiable, which can then be discussed in the municipal health conferences of administrative and municipal districts.

## Structure and methodology of the health barometer

**Figure 1:**
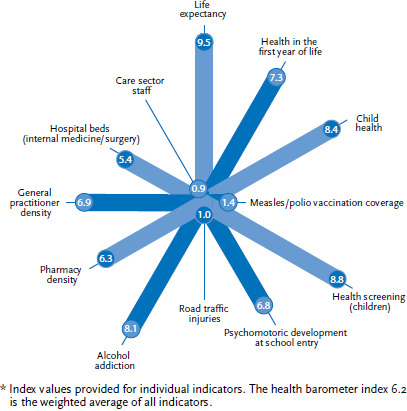
Example of a chart displaying indicators: health barometer index 6.2* Source: Mannheim Institute of Public Health, Social and Preventive Medicine [4]

The health barometer [4] consists of twelve indicators which can be grouped into four topical focuses: population health, health promotion and disease prevention, health behaviour as well as out- and inpatient care. The weighting of individual indicators as well as the selection of indicators influence the overall result of the health index. As a general indicator, the health barometer reflects health care efficiency within a particular district in Baden-Württemberg in comparison to others on a scale of 0 to 10. Health barometer values are calculated as a weighted average of individual indicators; these are composed of the four topical focuses mentioned above. The health barometer is calculated through a multi-step procedure. Initially, the underlying raw data is imported and used to create a single dataset. In a second step, based on this raw data, the variables that will serve as a basis for the utilised indicators are extracted. Following this, all variables are transformed into a uniform scale and correspondingly adjusted before the indicators are calculated. A higher value on this scale translates into a more positive expression of the indicator (for example, higher life expectancy or fewer road traffic deaths). The scale ranges from 0 (lowest value or worst district) to a maximum of 10 (best value or district). The district with the lowest score in a specific indicator is given 0 and districts beyond a 95 percentile receive 10. The health barometer is then calculated as a weighted average of the individual indicators (Figure 1).
